# Partial delignification of wood and membrane preparation using a quaternary ammonium ionic liquid

**DOI:** 10.1038/srep42472

**Published:** 2017-03-07

**Authors:** Jiaojiao Miao, Yongqi Yu, Zeming Jiang, Lan Tang, Liping Zhang

**Affiliations:** 1 MOE Key Laboratory of Wooden Material Science and Application, College of Materials Science and Technology, Beijing Forestry University, Beijing 100083, PR China

## Abstract

This work determined that southern yellow pine wood can almost be completely dissolved in the quaternary ammonium ionic liquid tetrabutylammonium acetate with dimethyl sulfoxide (in a 2:8 mass ratio), after minimal grinding, upon heating at 85 °C for three dissolution/reconstitution cycles, each 1.5 h. Approximately 34.6% of the native lignin and 67.4% of the native carbohydrates present in the original wood can subsequently be extracted, respectively, and were assessed. A gradual decrease in lignin with increased extraction cycles resulted in increased crystallinity index of the cellulose II in the cellulose-rich residue, as confirmed by X-ray diffraction. An increasingly homogeneous macrostructure in the cellulose-rich residue was also evident from scanning electron microscopy images. Membranes cast directly from either wood or cellulose-rich residue solutions in the same tetrabutylammonium acetate/dimethyl sulfoxide system, were prepared using a papermaking-like process. Morphological and mechanical studies indicated that lignin extraction made the membranes more uniform and flexible. Systematic increases in the fibril lengths and orientations of the recovered materials were also found with decreasing lignin contents on the basis of atomic force microscopy analysis. This work demonstrates that relatively efficient partial separation of pine wood and subsequent membrane preparation are possible using a quaternary ammonium ionic liquid.

Wood is among the most important biorenewable resources, and has many uses, including its application as a fuel and a building and manufacturing material. Highly transparent wood composite, taking advantage of the unique microstructure in wood, also has been obtained and could be used as structural materials in automobiles and optoelectronics in the future^1^. In addition, cellulose, hemicelluloses and lignin, the three major constituent biopolymers of wood, are widely used in other applications (Supplementary Fig. S1). Cellulose and hemicelluloses are applied in paper-making, textile, packing materials, and so on, while most of lignin is burned as a source of energy^2^,^3^.

Fortunately, recent research has also focused on the use of lignin as a raw ingredient for various chemicals, for example, dispersants, binders and emulsifiers^4^, or in the new synthesis processes of biopolymers^5^,^6^, biocomposites^7^,^8^,^9^, and biofuels^10^, as well as a source of carbon fiber^11^. However, the separation of lignocellulosic materials from wood and the processing of this material are difficult and energy-intensive. This difficulty results from the complex carbohydrate matrices inside the plant cell walls and the high mechanical strength that lignin imparts to these walls, both of which make the dissolution of wood challenging.

At present, the Kraft pulping method^12^ is the most widely used. In this process, the lignin/hemicellulose matrix in the wood is degraded by treatment with sodium hydroxide and sodium sulfide solutions at high temperature and pressure. Compared with other methods, higher yields of cellulose can be obtained via the Kraft technique. Despite its popularity and efficiency, the Kraft process has several disadvantages, primarily the potential for significant pollution owing to the variety of toxic and hazardous chemicals associated with this method. Newer approaches to fractionating biomass using acids^13^,^14^ under mild conditions have also been reported, but these also have limitations in terms of solvent recovery and the use of corrosive acids.

Recently, ionic liquids (ILs) have been investigated in a range of biomass processing applications as potential “green” solvents, due to their negligible vapor pressures and ready recoverabilities. Interest in using ILs as biomass solvents has so far been centered on the dissolution and processing of pure cellulose. In addition to imidazolium-based ILs, various quaternary ammonium ILs have been reported as solvents for cellulose, including tetrabutylammonium fluoride trihydrate (TBAF•3H_2_O)^15^, tetraethylammonium chloride (TEAC)^16^, and 40% tetrabutylammonium hydroxides (TBAH)^17^ with a cosolvent (Supplementary Fig. S1). Not only cellulose but also lignin can be dissolved in imidazolium ILs^18^ and so wood dissolution via some ILs has been studied and promising results have been obtained, showing that wood (including hardwood and softwood) can be partially dissolved, even completely, in these compounds^19^,^20^,^21^. To date, the ILs of choice for the dissolution and delignification of wood have been imidazolium ILs, including 1-butyl-3-methylimidazolium chloride ([C_4_min]Cl), 1-allyl-3-methylimidazolium chloride ([Amim]Cl), 1-ethyl-3-methylimidazolium acetate ([C_2_min]OAc), and 1-butyl-3-methylimidazolium acetate ([C_4_min]OAc)^19^,^20^,^21^. The 5% (w/w) of wood is dissolved in imidazolium ILs at a high temperature (>100 °C) with long time (8–24 h)^20^ and yield of recovered lignin is up to 5% (w/w) by one dissolution-reconstitution cycle^22^,^23^. However, these studies based on quaternary ammonium ILs have never been reported and the dissolution-reconstitution process has never been cycled.

A solvent system composed of tetrabutylammonium acetate (TBAA) and dimethyl sulfoxide (DMSO) has been reported to dissolve cellulose rapidly, at or near room temperature^24^. This occurs because the oxygen and hydrogen atoms of the cellulose form electron donor-acceptor (EDA) complexes with the charged IL species. The presence of the aprotic cosolvent DMSO also assists by partially breaking down the ionic association of the IL through solvation of its cation and anion constituents. The inter- and intramolecular hydrogen bonding in cellulose is disrupted and results in the dissolution of cellulose which is one of the main components of wood. As we excepted, southern yellow pine wood can almost be completely dissolved in TBAA/DMSO (in a 2:8 mass ratio), after minimal grinding, upon heating at 85 °C for three dissolution/reconstitution cycles, each 1.5 h. Compared with other methods of wood dissolution in ILs (mentioned before), the method above is more efficient due to lower temperature and less time.

In present work, we therefore utilized the TBAA/DMSO to dissolve wood powder directly. In addition to achieving the dissolution of wood, the lignin, as a major biopolymer fraction, was partially separated by the use of appropriate reconstitution solvents. The process, dissolution-extraction-reconstitution, was cycled three times. The residual cellulose-rich materials from each dissolution-reconstitution cycle were compared and used to prepare membranes. And the morphologies and mechanical properties of these membranes were studied.

## Material and methods

### Materials

The trials in this work used air-dried southern yellow pine wood (Mion Wood Co., Shanghai, China), ground to a fine powder (less than 0.45 mm particle size or less than 0.12 mm). Pure microcrystalline cellulose (MCC, Sinopharm Chemical Reagent Co., Shanghai, China) powder with a degree of polymerization (DP) of 220 was also employed. TBAA (>90%) was purchased from TCI (Japan) and used without further purification. All other chemical reagents were obtained from commercial sources in China and were analytical grade.

### Wood separation experiments

**Figure 1:**
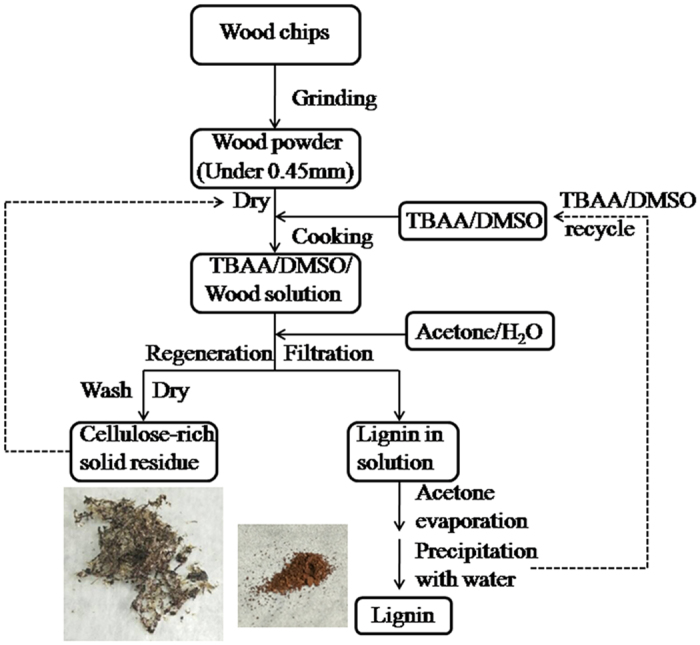
Flowchart for the dissolution and regeneration of wood in TBAA/DMSO.

The wood separation process used in this study is briefly described in Fig. 1. A quantity of wood powder (1.0 g, less than 0.45-mm particle size) was added to 20 g of a 2:8 (by mass) TBAA/DMSO mixture, after which the dispersion was rapidly heated to 85 °C in an oil bath (preventing the moisture in air into the mixture) and held at this temperature with stirring for 1.5 h. Subsequently, 50 ml mixed solution of acetone and water (equi-volume) was added to the cooled brown-colored wood solution, followed by centrifugation at 2800 × g for 10 min. The precipitated cellulosic materials were repeatedly washed with deionized water, followed by vacuum filtration through a nylon filter paper in a ceramic Büchner funnel. The acetone in the supernatant was evaporated in air at 80 °C and the remaining aqueous solution (and solid) was poured into deionized water (250 mL) to precipitate the lignin. The recovered lignin was separated by vacuum filtration and washed repeatedly with deionized water to ensure that all residual TBAA/DMSO was removed. The second and third lignin extraction cycles followed the same methodology. All the products (lignin and the thrice extracted cellulosic material) were subsequently freeze dried.

### Preparation of membranes made of wood and cellulose-rich residue

**Figure 2:**
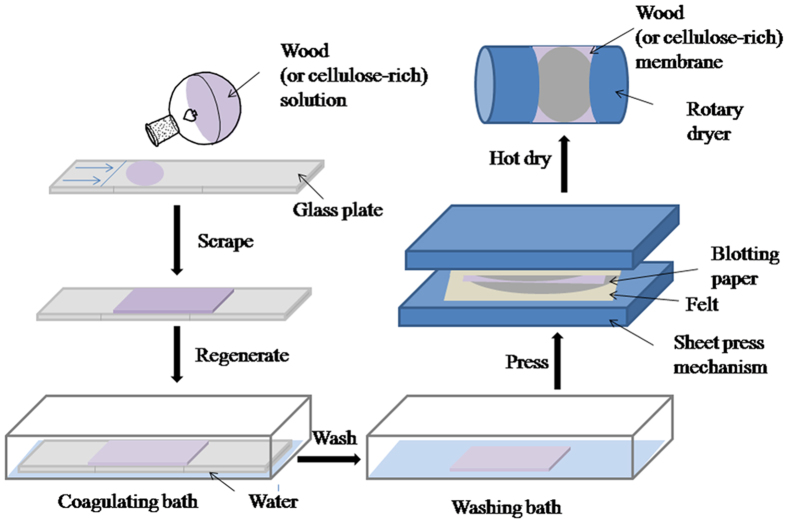
The process used to prepare wood or cellulose-rich residue membranes.

The preparation of membranes from wood or cellulose-rich extraction residue is briefly summarized in Fig. 2. In this process, 0.5 g of the cellulose-rich residue from each dissolution-reconstitution cycle (as described in Section 2.2) was added to 10 g of the TBAA/DMSO mixture (2:8 by mass) with stirring at 85 °C in an oil bath for 1.5 h. Because wood powder with particles 0.45 mm in size was found to be too difficult to dissolve completely, 0.5 g of wood powder with a 0.12-mm particle size was added to 10 g of the TBAA/DMSO mixture with stirring at 120 °C in an oil bath for 16 h. In this manner, clear wood or cellulose-rich residue solutions were obtained. These solutions were purged of bubbles by applying a 0.1-MPa vacuum and each solution was then poured onto a clean glass plate. The plates were manually scraped to form lamellar domains, using a custom fabricated drawknife, after which the solvent was allowed to evaporate in ambient air for 10 s and the plates were immersed in a water bath (100 ml) for 5 min. The resulting milky films were immersed in a second water bath (1000 ml) for three sessions, each one hour in duration, to ensure that any residual TBAA/DMSO was removed. It is worth noting that the edges of the cellulose sheets exhibited a tendency to curl if the film was immediately dried at a high temperature. For this reason, the process used to dry the resultant films were similar to those used during the papermaking process. Using this approach, each moist film was sandwiched between two sections of blotting paper and the resulting stack was then placed between two pieces of felt, after which the entire assembly was placed under a 0.41-MPa vacuum and pressed twice, applying a two min duration each time (KRK No. 2569 sheet press, Japan) to remove the majority of the water. Each film, still sandwiched between the blotting paper, was further dried at 105 °C for 1 h using a rotary dryer (KRK No. 2575–1, Japan) to obtain smooth, clear membranes.

### Dissolution of wood and cellulose-rich residue in TBAA/DMSO

In a typical dissolution trial, wood powder or cellulose-rich residue was added to 20 g of TBAA/DMSO mixture (2:8 by mass) in a round flask. Then the round flask was placed in an oil bath and heated on a hot plate with stirring while open to the atmosphere. The temperature at which dissolution occurred was monitored by a temperature control system in the oil bath. After heating, the solution was separated by centrifugation with high speed (100 g × 10 min) from the settled residue. The solid residue was washed with 200 ml deionized water and captured via vacuum filtration, dried overnight in an oven at 90 °C and then weighed. The percentage of wood or cellulose-rich residue dissolved was calculated according to the method of Sun *et al*.^25^:


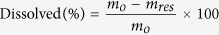


where *m*_*o*_ is the mass of the original sample (wood or cellulose-rich residue) and *m*_*res*_ represents the mass of the recovered residue.

### Characterization

FT-IR spectra of samples were recorded using a VERTES TOV spectrometer (Bruker, Germany) over the wavelength range of 4000–650 cm^−1^ at a resolution of 8 cm^−1^. Each sample was mixed with potassium bromide and formed into a pellet for analysis.

The recovered lignin was analyzed by ^13^C and ^1^H nuclear magnetic resonance (NMR) spectroscopy using a Bruker Avance 500 spectrometer. The lignin samples were dissolved at a concentration of 5% (w/w) in DMSO-d_6_ and spectra were recorded at 25 °C.

Elemental analysis was performed by Vario EL III (Elementar, Germany) to determine the carbon, hydrogen, and nitrogen content in the recovered lignin samples. Oxygen content was obtained by subtraction method. The methoxyl attached to the lignin was determined by ^1^H-NMR^26^.

The X-ray diffraction (XRD) analysis of samples was performed in the reflection mode using an XRD-6000 diffractometer (Shimadzu, Japan). Patterns were acquired with Cu Kα radiation (λ=1.5406 Å) at 40 kV and 30 mA over the 2θ range from 5 to 40° at a scan speed of 0.2° min^−1^. The crystallinity index (χ_c_) of each specimen was determined from the ratio of the separate crystalline peak area to the total reflection area including background.

Thermogravimetric analysis (TGA) was performed with a STA 449F3 instrument (NETZSCH, Germany). Each sample was heated from 50 to 600 °C at a rate of 10 °C·min^−1^ under argon.

The lignin was also assessed by differential scanning calorimetry (DSC) with a Q-500 instrument (TA Instruments, USA). DSC analysis was performed at a constant heating rate of 10 °C min^−1^ from 25 to 250 °C under a 25-ml min^−1^ N_2_ flow.

The morphologies of samples were examined by scanning electron microscopy (SEM). Cross-section specimens were prepared by fracturing the membranes in liquid nitrogen to avoid structural deformations of the materials. All samples were sputtered with Au or Pt (for the observation of nanopaticles) to obtain conductive specimens and to avoid degradation. SEM images were acquired at 1000 and 2000× magnification using a HITACHI S-3400N (Japan) operating at 5 kV and at 40000 and 80000× magnification using a JSM-7001F (Japan) operating at 20 kV.

The surface morphologies of the membranes were examined using atomic force microscopy (AFM, Multimode 8, Bruker, USA). The mica substrate was attached to a specimen holder and analyzed with tapping-mode AFM. During this process, RTESPA silica cantilevers (with a tip radius of 8 nm and a spring constant of 40 N m^−1^) were oscillated at a frequency of 300 kHz. Topographic (height) and phase images were recorded under controlled air conditions (23 °C and 50% relative humidity).

The tensile strength and elongation at break of wood (or cellulose-rich) membranes were determined using a computer-controlled tensile test instrument (ZB-WL300, Hangzhou Zhibang Automated Instrument Co., Ltd., China) at a strain rate of 10 mm/min. The apparent density was calculated by using basis weight (the weight per unit area) and the apparent thickness. The basis weight and apparent thickness were determined using TAPPI standard T410^27^ and TAPPI Method T411^28^, respectively. To minimize experimental error, each type of specimen was assessed five times.

Water contact angle data were obtained using a HARKE-SPCA apparatus (Harke, China). In preparation for these measurements, a membrane was attached to a glass slide with double-sided foam tape. The contact angle of a drop of deionized water (0.16 mL) on the surface of this film was subsequently determined approximately 3 s after application. To minimize the experimental error, the median of three tests is reported.

## Results and Discussion

### Dissolution of wood powder and cellulose-rich residue

The data in Supplementary Table S1 indicate that the smaller the wood particles the easier to dissolve in TBAA/DMSO. The smaller wood particles are obtained by the increased mechanical pulping which breaks down the internal structure and increases the surface area. In the case of the wood powder with a particle size of 0.45 mm, only 21.7% could be dissolved in TBAA/DMSO at 85 °C over 1.5 h. We therefore envisaged if, given a sufficiently high temperature and enough time, TBAA/DMSO could completely dissolve southern yellow pine. It was determined that 1.0 g of wood particles with a size of 0.45 mm could almost be completely dissolved in 20 g of the TBAA/DMSO mixture at 85 °C over 48 h. While [Amim]Cl could dissolve up to 5% (w/w) of Norway spruce sawdust at 80 °C within 24 h^20^. Another important variable is the temperature, which from 80 to 130 °C, has been reported in previous works^19^,^29^ about wood dissolution in ILs. Herein, complete dissolution of the pine (particle size of 0.45 mm) was observed at 120 °C after only 16 h. A high temperature and a prolonged dissolution evidently assist the dissolution process, although these conditions also result in increased degradation of the cellulose and greater energy usage.

During our experiments, we found that heating the wood for 1.5 h generated a gelatinous precipitate that would not completely dissolve in the TBAA/DMSO. Interestingly, after washing and drying, this material (representing the first recovery of cellulose-rich material) was readily dissolved upon heating in TBAA/DMSO at 85 °C for 1.5 h. This process evidently resulted in a loosened wood structure, and partial removal of both lignin and cellulose. Compared with one dissolution-reconstitution cycle in TBAA/DMSO or other ILs (discussed above), increasing the cycle number, considered in this work, can shorten the wood dissolution time and temperature. The dissolution of the second batch of recovered cellulose-rich material in TBAA/DMSO only required 30 min at 45 °C. As a final test, we recovered lignin and cellulose-rich material from the wood powder with a particle size of 0.45 mm following each dissolution-reconstitution cycle, then analyzed these materials. Each dissolution cycle involved heating the wood at 85 °C for 1.5 h.

### Quantification of carbohydrates and lignin recovered from wood powder

**Figure 3:**
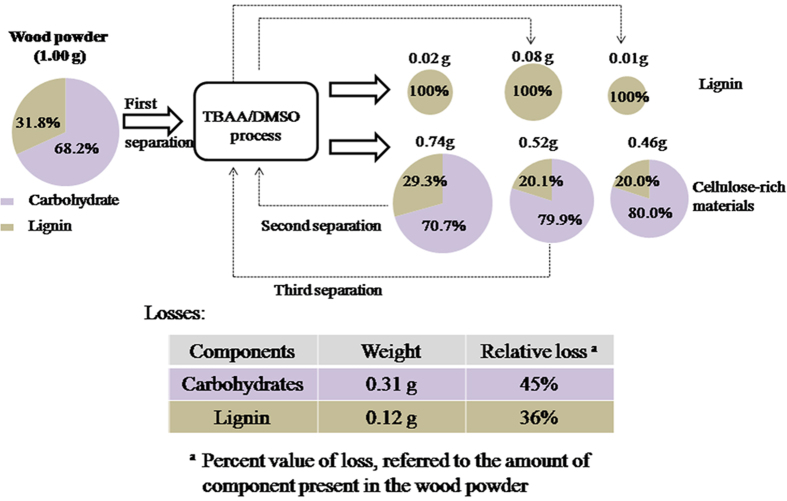
Mass balance for the total dissolution of 1.0 g of southern yellow pine wood (particle size under 0.45 mm) in 20 g of TBAA/DMSO with subsequent recovery of materials.

The composition and mass of the products by three extraction cycles were analyzed. The weight of the original wood powder was 1.0 g. The mass balance for this system is shown in Fig. 3. The lignin contents (including acid soluble lignin and Klason lignin) of the wood and cellulose-rich materials were measured by the TAPPI UM250 (1991)^30^ and TAPPI 222 (1998) methods^31^. It was found that only 0.012 g (or 1.2% of the original wood sample) of lignin was obtained by precipitation from deionized water; whereas an additional 0.008 g of lignin (0.8% of the original wood sample) was recovered upon adjustment to a pH range of 2 to 3 using HCl, for a total of 0.02 g (2.0% of the original wood) recovered lignin from the first cycle. Upon complete dissolution of the first batch of recovered cellulose-rich material, 0.05 g (6.7% of the cellulose-rich residue) of lignin was obtained by evaporating the acetone in the second cycle, and 0.03 g (4.0% of the cellulose-rich residue) was recovered upon adjusting the pH to the range of 2 to 3, for a total of 0.08 g (10.8% of the cellulose-rich residue). This lignin was also completely free of carbohydrates. The third cycle resulted in a small amount of additional delignification, in which the cellulose-rich residue from the second cycle was used. Overall, 34.6% of the initial lignin (31.8% of the original wood) was recovered as carbohydrate-free material, and 67.4% of the initial carbohydrates (68.2% of the original wood) were obtained as cellulose-rich material, in which 28.9% of the initial lignin remained bonded. The remaining 36.5% of the initial lignin available in the wood was lost, mainly because of washing steps in the current process.

### Analysis of the recovered lignin

#### Lignin molecular structure

A detailed elucidation of the structural features of the lignin was performed using various spectroscopic techniques (FT-IR and ^1^H- and ^13^C NMR), and representative spectra are shown in Supplementary Fig. S2. The FTIR spectrum of the lignin (Supplementary Fig. S2a) is dominated by a wide band from 3440 to 3200 cm^−1^, attributed to phenolic and aliphatic OH groups, followed by a methyl and methylene C-H stretching peak at 2926 cm^−1^, a methoxyl C-H stretch at 2854 cm^−1^, unconjugated C=O stretch at 1722 cm^−1^, aromatic skeleton C-C stretch at 1650–1480 cm^−1^, methyl and methylene asymmetric C-H deformations at 1460 and 1400 cm^−1^, and C-O (H) and C-O (C) stretching of primary aliphatic OH and ether groups at 1033 cm^−1^ 22,23,32. This spectrum is in excellent agreement with the reference spectrum of Indulin purchased from Sigma Aldrich^22^. In the case of the ^13^C-NMR spectrum in Supplementary Fig. S2(b), the majority of the signals can be attributed to lignin, including the aromatic carbons at 155–100 ppm, the aliphatic side chain carbons at 86–60 ppm, and the methoxyl carbons at 55 ppm, although there is a lack of resolution^33^. It was worth mentioning that some content of carbohydrates was contained in the recovered lignin as evidenced by the presence of signals at 101.6, 76–73, and 20.9 ppm corresponding to C-1 of xylan, C-2 ~ C-4 of xylan, and acetyl CH_3_, respectively^33^. Furthermore, the ^1^H-NMR spectrum in Supplementary Fig. S2(c) exhibits a peak in the chemical shift range from 7.2 to 6.5 ppm, assigned to aromatic H, and a peak at 4.2 to 3.1 ppm attributed to methoxyl groups, both corresponding to the ^1^H chemical shifts expected from lignin^34^. Moreover, the lignin has the C_900_ unit formula C_900_H_815_O_301_N_6_(OCH_3_)_118_ (Supplementary Table S2), which was calculated by Elemental analysis and ^1^H-NMR. And the nitrogen in the recovered lignin mainly resulted from the ionic liquid TBAA used in this work, which has one mole of nitrogen per IL molecule.

#### Thermal behavior analysis of recovered lignin

The thermogravimetric and differential thermal analyses (TGA/DTG) data for the recovered lignin from solutions of pine wood in TBAA/DMSO are displayed in Supplementary Fig. S3(a). The DTG plot shows that the thermal decomposition of the material proceeds in four major steps. Step I, below 150 °C, involves the dehydration of lignin, and depends on the amounts of water and low molecular weight volatiles in the lignin^35^,^36^. The maximum mass loss rate in step I (T_m1_) appears at approximately 90 °C. The maximum weight loss rate in step II (T_m2_) was at 264 °C, although there is little change observed in the TG curve (Supplementary Fig. S3a); this step is associated with the rupture of C-C bonds^37^. In Step III, initial decomposition takes place, which involves fragmentation of inter-unit linkages in the lignin^37^, with coke, organic and phenolic compounds, and gaseous substances as the main products. The maximum weight loss rate in Step III is observed at 351 °C. In Step IV, above 400 °C, the final decomposition of the aromatics components of the lignin occurs^38^.

The DSC plot of the lignin extracted from the pine is displayed in Supplementary Fig. S3(b). The glass transition temperatures (T_g_) of the lignin can be determined from these curves, and appears at 93 °C. Previously, T_g_ values of 86 to 88 °C and 108 °C have been reported for lignins from solutions of pine woods in imidazolium ILs (AmimCl, [C_4_mim]Cl and [C_2_mim]Cl) and Indulin, respectively^39^. The results of the present study are also similar to those reported elsewhere in the literature, including T_g_ values between 85 and 180 °C^34^,^37^,^40^. The differences in these T_g_ values may be due to the varying thermal histories and molecular weight distributions of the lignin samples^41^.

### Analysis of the cellulose-rich residue

#### The cellulose-rich residue molecular structure

The FT-IR spectra obtained from the original pine wood powder and the recovered cellulose-rich materials are compared with that of pure MCC in Supplementary Fig. S4. The spectra of the cellulose-rich residues recovered from various cycles are similar to that of the MCC, and all the expected bond vibrations, including O-H, C-H and C-O, are observed. The O-H vibration between 3500 and 3200 cm^−1^ appears as a strong, broad absorption, indicating hydrogen bonding of the O-H group, which indeed takes place between repeating units within the cellulose matrix. The C-O vibration appears as a strong absorption between 1150 and 1000 cm^−1^, indicating that the recovered material was enriched in carbohydrates. The C-H vibration is also evident at 2920 cm^−1^. Although typical lignin peaks, such as methoxyl C-H stretching at 2854 cm^−1^ and aromatic skeleton C-C stretching at 1650–1480 cm^−1^, are also present in the spectrum of the cellulose-rich material (indicating the presence of lignin), the intensity of these peaks is reduced as the extraction times are increased, especially when comparing the original wood powder to the second cellulose-rich residue sample (spectra 4a and b2). These results indicate that the lignin was gradually removed. In contrast, the spectra of the second and third cellulose-rich residues are similar. Therefore, three extraction cycles are sufficient to remove as much lignin as possible when employing TBAA/DMSO, a result that is further confirmed by XRD spectra.

#### XRD analysis of the cellulose-rich residue

**Figure 4:**
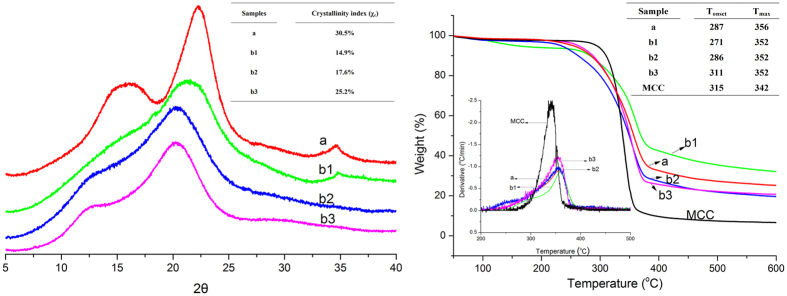
XRD spectra (left) and TG/DTG curves (right) of (a) wood powder, and cellulose-rich residue obtained following the (b1) first, (b2) second, and (b3) third extraction cycles.

XRD experimental spectra in Fig. 4 were obtained from the wood powder and the cellulose-rich materials generated from each of the three extractions. And respectively deconvoluted spectra were analyzed using *Jade 5.0* (Supplementary Fig. S5). In the case of the wood powder, the cellulose was present in its native form, and so produced a cellulose I pattern, with characteristic diffraction maxima at 2θ = 14.8°, 16.5°, 22.3° and 34.6°, associated with the (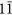
0), (110), (200) and (400) planes, respectively. The typical cellulose II pattern contains peaks at approximately 12.3°, 20.1° and 22.1°, associated with the (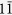
0), (110), and (002) planes. The crystalline structures of the second and third cellulose-rich residues appear to match this same pattern (spectra b2 and b3), although (110) and (002) peaks overlap at 20.3° (Supplementary Fig. S5). In the case of the first cellulose-rich residue, both cellulose I and cellulose II appear to coexist due to the incomplete dissolution of the wood powder in the first cycle (Supplementary Fig. S5). The crystallinity index values (χ_c_) of the cellulose-rich residues from the first, second and third cycles were 14.9, 17.6 and 25.2%, respectively. In the case of the wood powder, the crystal conversion from cellulose I to cellulose II either does not occur or takes place with difficulty because a matrix substance, such as lignin, prevents the interdigitation of cellulose microfibrils during the recovery process^42^,^43^. In purified pulps, the cellulose I structure will swell, such that the molecular chains move apart in the cellulose solvent system. During this process, cellulose chains of opposite polarity can intermingle, such that cellulose II is generated upon the removal of solvent^43^. Thus, with increasing numbers of dissolution-reconstitution cycles, the lignin was gradually removed from the wood, cellulose II was formed more readily and χ_c_ increased. After regeneration, cellulose II was recovered, which was proof of the true dissolution of the wood in TBAA/DMSO.

#### Thermal behavior of the cellulose-rich residue

TGA/DTG analysis of the original wood powder, cellulose-rich residue and pure MCC generated the plots shown in Fig. 4. The maximum decomposition temperatures (T_max_) of each of the three cellulose-rich residues were similar, at approximately 352 °C, while the degradation onset temperature (T_onset_) was increased with increasing cycles. This is attributed not only to the decrease in lignin but also the incremental increases in the degree of crystallinity. The T_onset_ of the final batch of cellulose-rich material was 311 °C, similar to the value determined for the MCC. In comparison, the T_max_ of the this same material was 352 °C, 10 °C higher than that of the MCC and indicating a significantly higher degree of polymerization. From the first to the third cycle, the residue remaining above 600 °C was decreased due to the absence of lignin and ash content, which have a low degradation rate, such that the lignin residue was 35 wt%, as shown in Supplementary Fig. S3a. However, the thermal decomposition parameters of the original wood powder were comparable to those of the first and second cycle cellulose-rich materials, which had T_onset_ and T_max_ values of 287 and 356 °C, respectively, even though these had the highest levels of lignin. This result indicates variations in the morphologies and decreases in the DP values of the cellulose materials recovered from TBAA/DMSO-based wood liquors, which is agree with other paper^44^.

#### Morphologies of the cellulose-rich residues

**Figure 5:**
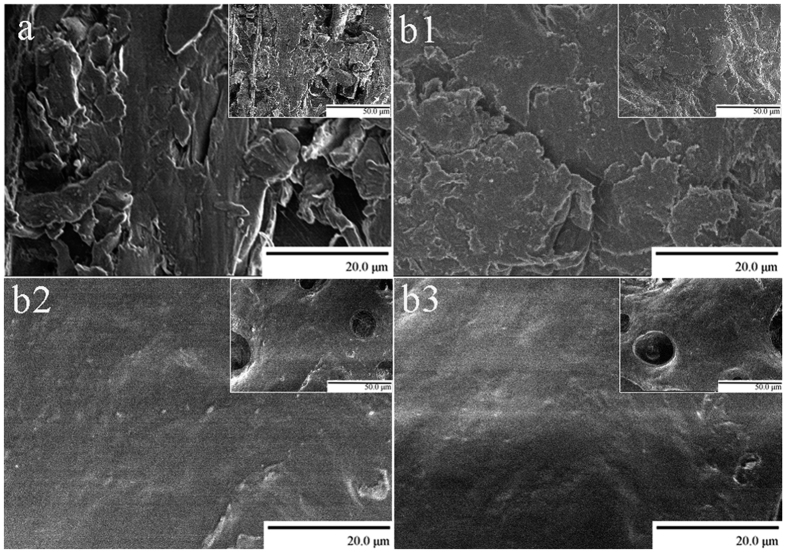
SEM images of (**a**) wood powder, and cellulose-rich materials after the (b1) first, (b2) second, and (b3) third cycles. Insets are the respectively less magnified images with scale bar of 50 μm.

The morphologies of the wood powder and the cellulose-rich residues are presented in SEM images (Fig. 5). At a higher magnification (2000×), the surface of the cellulose-rich material evidently becomes smoother as the cycles are repeated. Compared with the original wood fiber, with a conglomerate texture, the cellulose-rich residue, especially after the second and third cycles, exhibits a relatively homogeneous macrostructure. This phenomenon is primarily attributed to the reduction of lignin, which prevents the aggregation of cellulose microfibrils during regeneration.

### Analysis of cellulose-rich membranes

#### Morphology of the cellulose-rich membranes

**Figure 6:**
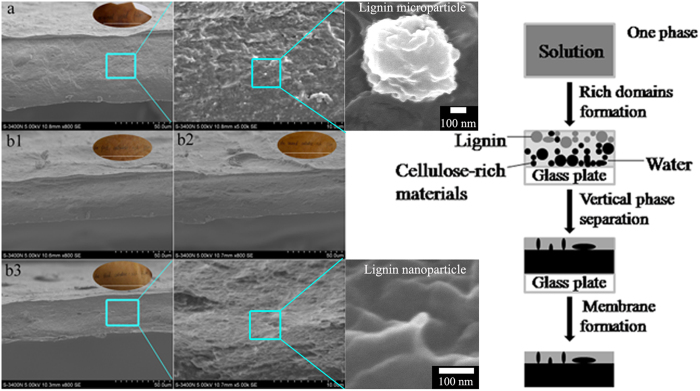
SEM images (left) of the cross-sections of membranes prepared from (a) wood powder, and cellulose-rich material from the (b1) first, (b2) second, and (b3) third cycles. Inserts: photographs of semitransparent membranes with a thickness of approximately 50 μm, labeled with the sample name. A schematic illustration (right) of layer formation during the membrane preparation.

As a final assessment of their mechanical properties, we explored the use of the regenerated samples in the preparation of cellulose-rich membranes, following the methods described in the Experimental section (Fig. 2). Briefly, 5 wt% solutions of either the original ground wood or the cellulose-rich residue re-dissolved in the TBAA/DMSO solvent system were prepared, cast onto glass plates as thin films, and perfused extensively with water. Casting membranes directly from the wood solution proved unsuccessful; these membranes were fragile (Fig. 6a), suggesting that the presence of large amounts of lignin and/or hemicelluloses inhibits the formation of structured cellulose hydrogels. However, the decreased level of lignin in the cellulose-rich residue resulted in better membrane formation and improved membrane flexibility (Fig. 6b1), although the membranes were also frangible. As the lignin content was decreased with each dissolution-reconstitution cycle, the membranes were found to take on a lighter coloration and to increase in transparency. The opacity of such membranes is closely connected to the surface and internal heterogeneity^45^, and this was further confirmed by SEM analysis of the membranes.

**Figure 7:**
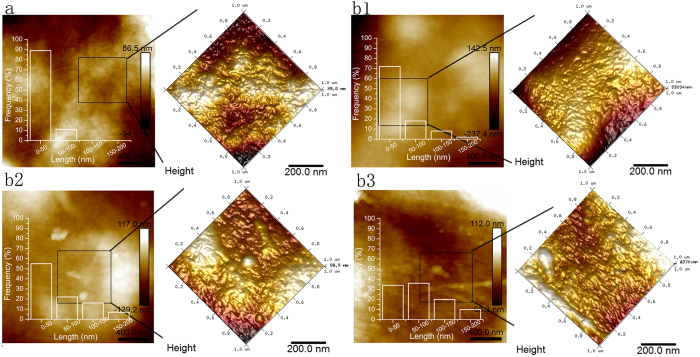
AFM height images of the membranes made from (a) Wood powder, and cellulose-rich material from the (b1) first, (b2) second, and (b3) third cycles. Two scan sizes are shown: 2 × 2 μm (2D) and 1 × 1 μm (3D). The respective length distribution of cellulose fibers in each AFM image are shown in the 2D pictures.

Cross-sections of the wood and cellulose-rich membranes were obtained and compared by SEM. Representative images are provided in Fig. 6. In the case of the wood membranes, a distinct thin layer near the surface was observed and the interior was evidently composed of microscopic, spherical particles with diameter of 0.3–0.6 μm, believed to be lignin (Fig. 6a). The microparticles existing in the SEM picture of the wood membrane disappeared in that of the third cellulose-rich materials (Fig. 6b3 middle), mainly because of the extraction and degradation of lignin during three dissolution/extraction cycles. And nanoparticles in the third cellulose-rich materials can be observed from the SEM picture with high resolution (Fig. 6b3 right), which was further confirmed by AFM (returning to Fig. 7b3). In the membrane prepared from wood powder, and cellulose-rich materials from the first and second cycle, a distinct thin layer near the surface was observed and the thickness was 4.6 μm, 3.1 μm and 0.8 μm, respectively. The cellulose-rich material exhibited less obvious layer with lower lignin content^46^. It is apparent from Fig. 6(b3) that the internal structure of the final cellulose-rich membrane was quite uniform and that there were no obvious layers. An illustration summarizing one possible layer formation mechanism is provided to the right of Fig. 6, assuming that the blended polymers (cellulose, hemicelluloses and lignin) undergo similar phase separation mechanisms, as has been reported for derivatives of cellulose (TMSC) and lignin (lignin acetate)^47^. In this system, the polymers initially dissolve in the TBAA/DMSO as a single phase system. As the solvent is exchanged and the recovery process proceeds, different phase inversion rates, molecular weights and poor compatibility result in the formation of both lignin- and cellulose-rich phases.

The surface topographies of the membranes were evaluated by AFM using height images. Fibrils were evidently formed and are distinctly present in the 3D AFM images of each membrane (Fig. 7), even though the wood powder and each cellulose-rich material were completely dissolved in the TBAA/DMSO. The length distributions of the fibrils were obtained by AFM analysis employing the NanoScope Analysis software (Fig. 7). In the case of the wood membrane, the fibrils were curved and seemingly unordered, with weighted average lengths of 0–50 nm (Fig. 7a). However, in the membrane made from the third cycle cellulose-rich materials, the orientation of the fibrils was distinct and the lengths were much greater (with averages in the region of 50–100 nm, Fig. 7b3). We also observed a systematic increase in fibril length and orientation with decreasing lignin content. Cellulosic radicals are extremely reactive and can thus participate in recombination reactions via crosslinking. At higher lignin contents, the radical scavenging ability of the lignin, which is a known antioxidant, resulted in less pronounced crosslinking of the cellulose^46^. Another reason for these results might be that the cellulose-rich phase, in which the fibrils were longer and well oriented, was more highly exposed with decreases in the lignin-rich phase (Fig. 6, right). Some small, globular particles can be seen in the third cycle cellulose-rich membrane, especially in the 2D height image (Fig. 7b3). The diameter was 40–80 nm (Supplementary Fig. S6), which was too small to be found in SEM picture with low resolution (Fig. 6b3 middle). And similar particles were assembled and are clearly evident in the wood membrane (Fig. 7a). It is likely that these globular features correspond to lignin nanoparticles.

#### Water contact angles and tensile properties

**Figure 8:**
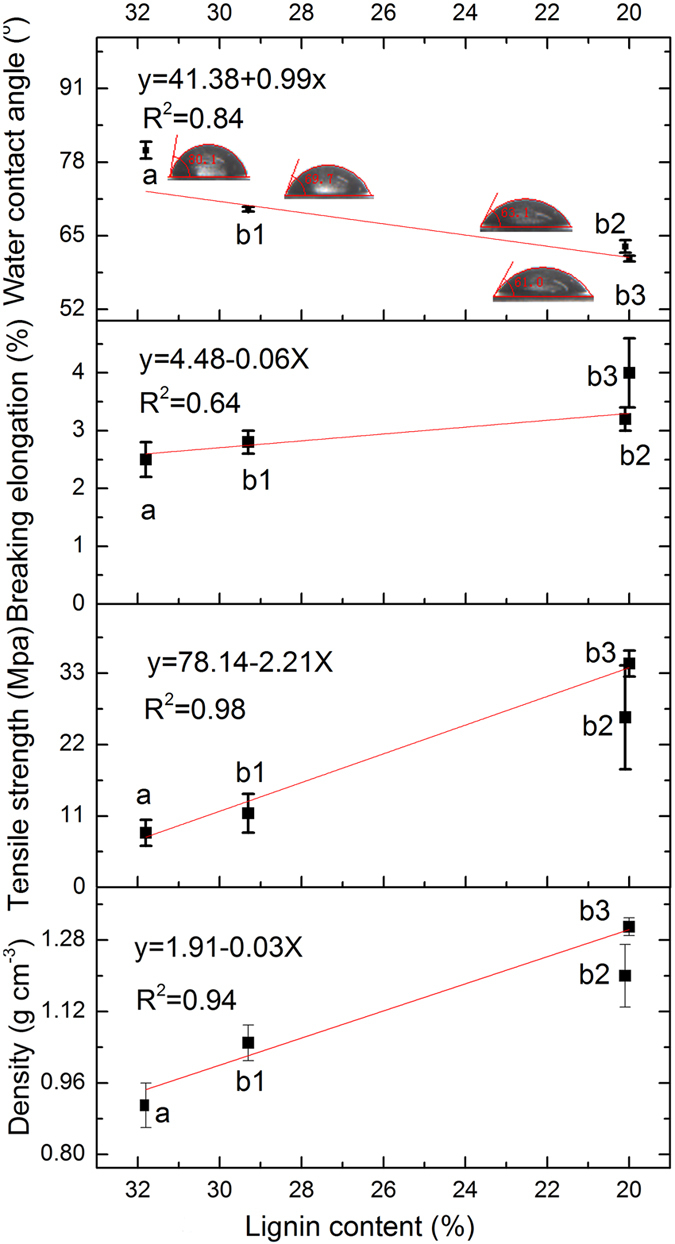
Ranges of values for density, tensile strength, elongation at break and water contact angle of membranes prepared from (a) wood powder, and cellulose-rich residue from the (b1) first, (b2) second, and (b3) third cycles.

It should be noted that the density and tensile properties (strain at break and tensile strength) increased with repeated dissolution-reconstitution cycles. The values measured for these properties were plotted against lignin content in the membranes, and the possible quantitative relation was derived in Fig. 8. The density increased with the reduced lignin content of the membranes. The fibrils in the membranes with decreasing lignin content are softer and thus more able to conform to each other, when the membranes are regenerated in the water bath^46^. Meanwhile, recall from the discussion of Fig. 6 that the membranes containing less residual lignin are more uniform and with reduced layer. These indicate that less lignin content in membranes results in a more compacted structure, in agreement with the density measurement. The increase in tensile strength with repeated dissolution-reconstitution cycles is consistent with the increased density (increased compaction) of membranes, because of the increased bonding capacity and collapsibility of the fibrils^48^. It is worth mentioning that the crosslinking of the longer cellulose fibrils in the membranes with decreasing lignin content (Fig. 7) is another important factor, attributing to the increased tensile strength. The increase in stain at break strain with the lignin content is likely related to the dispersion of components during the manufacture of the membranes.

The water contact angles (WCAs) were determined for membranes with different lignin contents. As shown in Fig. 8, the WCA decreased with reduction in the lignin content. The lignin decreased the surface energy and hydrophilic properties of the membranes, as would be expected based on the larger percentage of C-C and C-H bonds and the lower O/C ratio of lignin compared with cellulose^49^. In addition, the wetting properties of the solids will be magnified by the roughened surface^50^. For a water-wettable solid, the wettability will be further enhanced with increased roughness. All the membranes in this work exhibit water-wettable (WCA < 90°), in such cases wettability will be increased with an increasing roughness. The fibrils in the membranes with lower residual lignin content presented greater length and formed rougher surfaces (Fig. 7). The roughest surface (the membrane prepared from the third cycle cellulose-rich materials) showed strongest wettability (lowest WCA). Thus, the decrement in WCA value with the decreasing lignin content not only derived from the reduction in hydrophobic groups of lignin, but also arose from the increasing roughness of the membrane surfaces.

## Conclusion

A solvent system based on TBAA/DMSO was capable of completely dissolving southern yellow pine wood after minimal grinding. In general, smaller particle sizes, longer dissolution times and higher temperatures resulted in improved dissolution. Meanwhile, increasing the number of dissolution-reconstitution cycles can shorten the dissolution time and temperature. A portion of the lignin was extracted from the wood, as confirmed by analyses of structural and physical properties. Recovery of the wood solution from acetone/water (equi-volume) yields a cellulose-rich materials that are enriched in carbohydrates compared to the wood, and in which the content of lignin is gradually reduced over three extraction cycles. With the decrease in lignin, increasing thermal stability and χ_c_, in conjunction with the formation of cellulose II crystals, were identified in the cellulose-rich material recovered from each dissolution- reconstitution cycle. A detailed characterization of the products was performed to elucidate the effects of the lignin on the cellulose fibrils and membranes. The membranes with less lignin were found to be more uniform. A reduction in the lignin content lengthened the fibrils in the membranes and improved their orientation. The tensile properties and hydrophilic properties of the membranes were also promoted. Thus, it was possible to realize the partial separation and membrane preparation from wood components with relatively high efficiency and little degradation of natural polymers by using nontoxic chemicals.

Complete or at least improved separation of the constituent biopolymers via the TBAA/DMSO solvent system will be focused in future work, as well as the application of the cellulose-rich material, such as to obtain nanopaper. In addition, future research into efficient recycling of the solvent system will also be important. Further studies will focus on obtaining.

## Additional Information

**How to cite this article**: Miao, J. *et al*. Partial delignification of wood and membrane preparation using a quaternary ammonium ionic liquid. *Sci. Rep.* **7**, 42472; doi: 10.1038/srep42472 (2017).

**Publisher's note:** Springer Nature remains neutral with regard to jurisdictional claims in published maps and institutional affiliations.

## Supplementary Material

Supplementary Information
