# Supplementary material for: Partial delignification of wood and membrane preparation using a quaternary ammonium ionic liquid
Source: Sci Rep. 2017 Mar 7;7:42472. doi: 10.1038/srep42472 (PMC5339785; doi:10.1038/srep42472)
Supplement: Supplementary Information [file srep42472-s1.pdf]

Partial delignification of wood and membrane preparation using a quaternary ammonium ionic liquid

Jiaojiao Miao, Yongqi Yu, Zeming Jiang,Liping Zhang*

MOE Key Laboratory of Wooden Material Science and Application, Beijing Key Laboratory of Lignocellulosic Chemistry, College of Materials Science and Technology, Beijing Forestry University, Beijing 100083, PR China

Electronic Supplementary Information

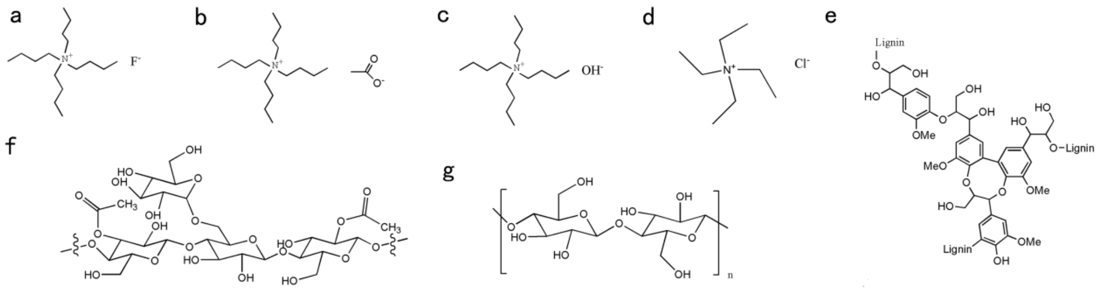

Fig. S1. Structures of the quaternary ammonium ILs referenced in this paper, as well as the three major constituent biopolymers of wood: (a) tetrabutylammonium fluoride (TBAF); (b) tetrabutylammonium acetate (TBAA); (c) tetrabutylammonium hydroxide (TBAH); (d) tetraethylammonium chloride (TEAC); (e) lignin; (f) structure of a representative hemicellulose (galactoglucomannan, the major hemicellulose in softwood); and (g) cellulose.

*Corresponding author. Tel.: +86-13911868160;
E-mail address: zlp418@163.com

Table S1 Percentage of wood powder dissolved and cellulose-rich material recovered from TBAA/DMSO[a] trials

Sample	Particle size	Temperature	Time	Dissolution
Original wood powder	≤0.12	120 ℃	16 h	100%
Original wood powder	≤0.45	85°C	48 h	92.7%
Original wood powder	≤0.45	120°C	16 h	91.5%
Original wood powder	≤0.45	85°C	1.5 h	21.7%
The first cellulose-rich residue	----	85°C	1.5 h	100%
The second cellulose-rich residue	----	45°C	0.5 h	100%

[a]The solvent system composed of the quaternary ammonium ionic liquid tetrabutylammonium acetate (TBAA) in conjunction with dimethyl sufoxide (DMSO) (in a 2:8 mass ratio).

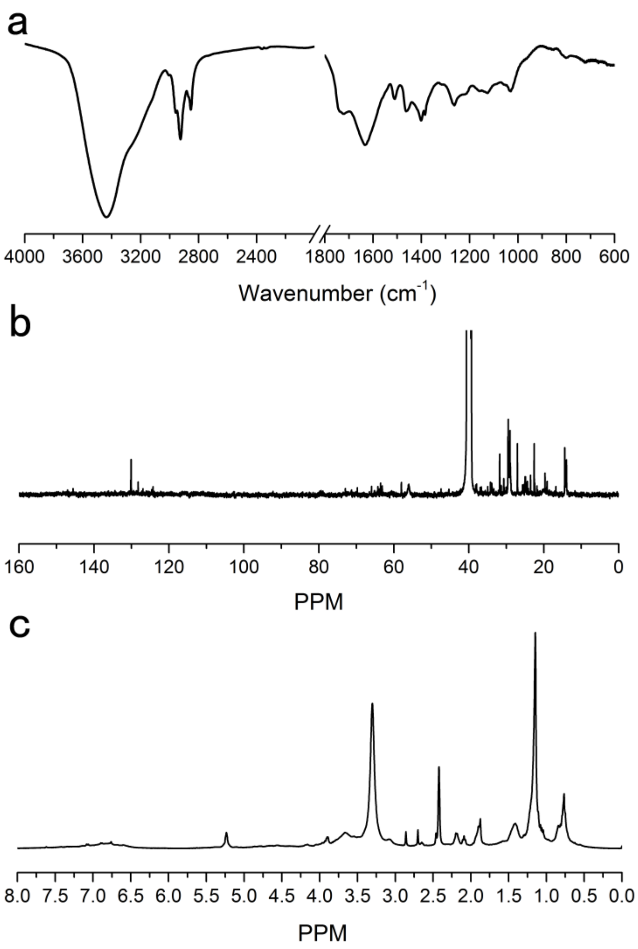

Fig. S2. Spectroscopic analyses of recovered lignin in DMSO-d_6: a) FT-IR, b) ^{13}C-NMR, and c) ^{1}H-NMR spectra.

Table S2 The weight content of the elements in the recovered lignin.

Element content	
C (%)	60.52
H (%)	5.79
O (%)	33.24
N (%)	0.45
OCH_3	18.41
C_{900} formula	$C_{900}H_{815}O_{301}N_6(OCH_3)_{118}$

C_{900} formula: $C_xH_yO_z N_\Omega (OCH_3)_n$

$$n = (\%\ OCH_3)/31.04;\ x = (\%C)/12 - n;\ y = (\%H) - 3n;\ z = (\%O)/16 - n;\ \Omega = (\%N)/14$$

$$\%\ OCH_3 = 28.28436 - 19.750047\chi$$

$$\chi = S_{H(aromatic)}/S_{H(methoxyl)}$$

where $S_{H(aromatic)}$ and $S_{H(methoxyl)}$ represent the areas of the peak in the chemical shift range from 7.2 to 6.5 ppm, assigned to aromatic H, and the peak at 4.2 to 3.1 ppm attributed to methoxyl groups in ^{1}H-NMR spectrum of the recovered lignin, respectively.

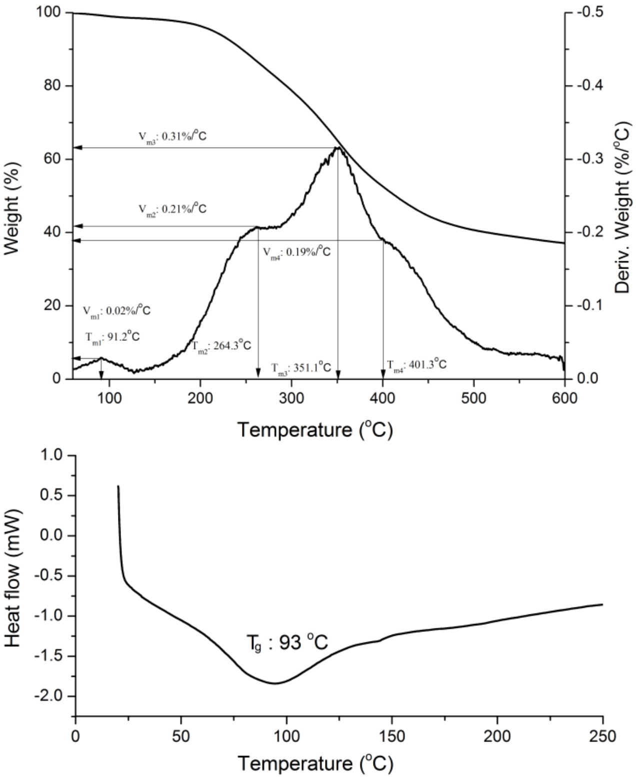

Fig. S3. (a) TGA/DTG and (b) DSC curves obtained from recovered lignin.

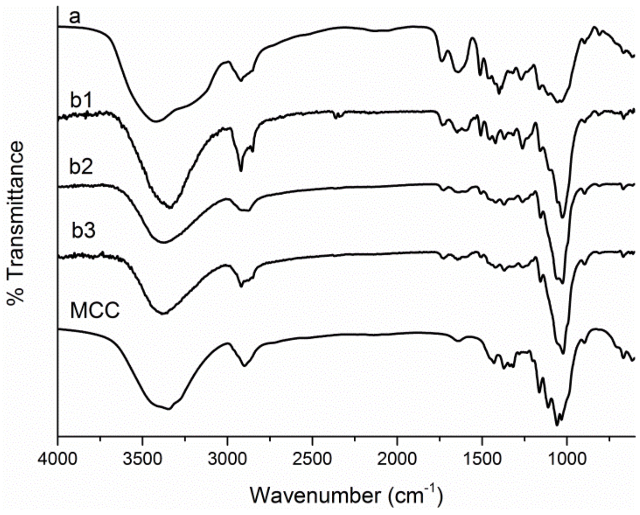

Fig. S4. FT-IR spectra obtained from MCC, (a) the original wood powder, and cellulose-rich residue following the (b1) first, (b2) second and (b3) third extraction cycle.

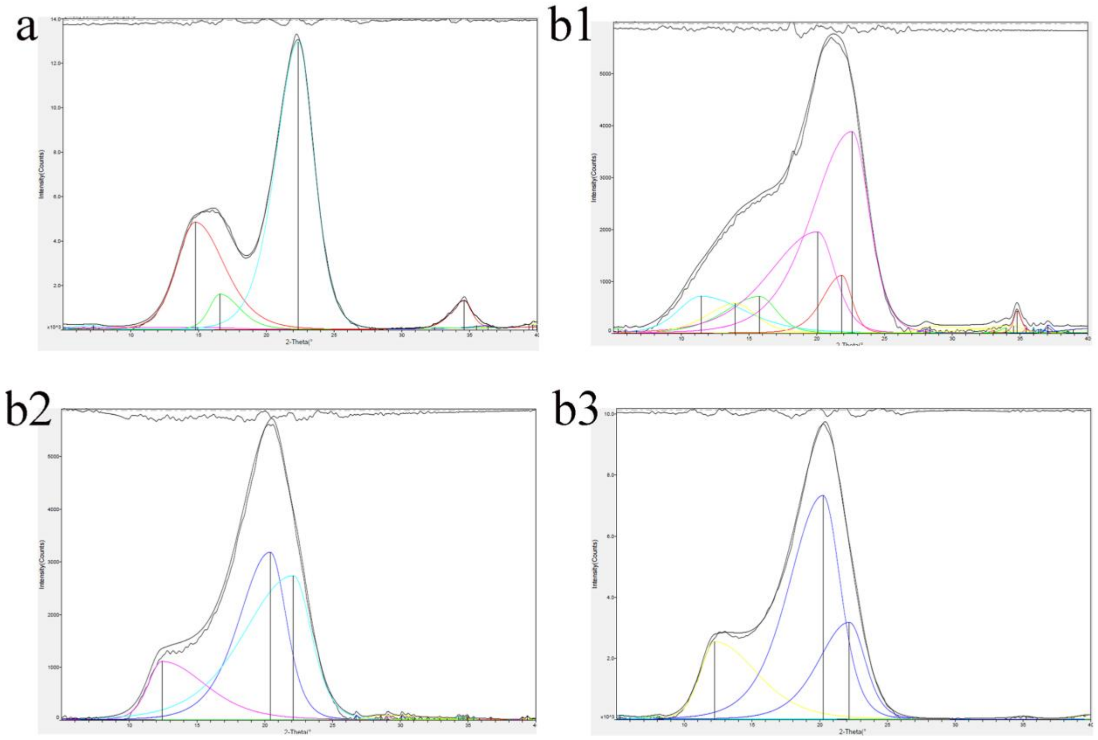

Fig. S5. The experimental and deconvoluted XRD spectra of (a) wood powder, and cellulose-rich residue obtained following the (b1) first, (b2) second, and (b3) third extraction cycles.

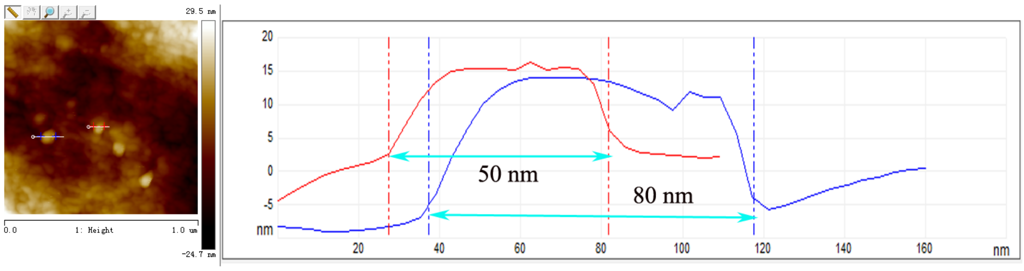

Fig. S6. Left: AFM height image of the third regenerated cellulose-rich materials with scan size $1 \times 1 \mu m^2$. Right: the height profile of the lignin particles in respective AFM height image.
